# Engaging healthcare students in innovative approaches for antimicrobial resistance containment

**DOI:** 10.4102/jphia.v15i1.645

**Published:** 2024-10-17

**Authors:** Hassan Kasujja, Henry Kajumbula, Jonans Tusiimire, J.P. Waswa, Stella M. Nanyonga, Reuben Kiggundu, Daniel C. Mwandah, Marion Murungi, Nathan Mugenyi, Irene M. Mukenya, Mohan P. Joshi, Dan Schwarz, Felix Bongomin, Niranjan Konduri

**Affiliations:** 1 USAID Medicines, Technologies, and Pharmaceutical Services Program, Management Sciences for Health, Kampala, Uganda; 2 Department of Microbiology, College of Health Sciences, Makerere University, Kampala, Uganda; 3 Faculty of Medicine, Mbarara University of Science and Technology, Mbarara, Uganda; 4 Education Committee, Pharmaceutical Society of Uganda, Kampala, Uganda; 5 Antimicrobial Resistance Committee, Pharmaceutical Society of Uganda, Kampala, Uganda; 6 Department of Pharmacology and Toxicology, School of Pharmacy, Kampala International University, Ishaka-Bushenyi, Uganda; 7 Department of Medical Microbiology and Immunology, School of Medicine, Kabale University, Kabale, Uganda; 8 USAID Medicines, Technologies, and Pharmaceutical Services Program, Management Sciences for Health, Arlington, United States of America; 9 Global Health Systems Innovation, Management Sciences for Health, Medford, United States of America; 10 Department of Medical Microbiology and Immunology, Faculty of Medicine, Gulu University, Gulu, Uganda

## Introduction

Antimicrobial resistance (AMR) occurs when organisms change over time and no longer respond to medicines making infections harder to treat.^1^ Antimicrobial resistance continues to impact global populations, increasing morbidity and mortality, straining healthcare systems and reducing countries’ gross domestic product.^2,3^ It is a leading cause of death worldwide, with an estimated 4.5 million associated deaths, which is expected to grow to 10 million deaths per year by 2050 if not responded to adequately.^3^ Furthermore, it is estimated that without adequate action, AMR could lead to a global reduction in gross domestic product of $3.4 trillion annually by 2030.^4^ For this reason, combating AMR is among the action packages of the Global Health Security Agenda.^5^ Although inappropriate use of antimicrobials is the leading driver of AMR, which is most evident in low-income countries, other factors, such as poor infection prevention and control and insufficient practices in water, sanitation and hygiene, contribute to the problem.^6,7^ According to the 2022 World Health Organization (WHO) Global AMR and Use Surveillance System report, Uganda had one of the highest antimicrobial consumption rates, with a defined daily dose of nearly 60 per 1000.^8^ Among the key drivers of inappropriate antimicrobial use and poor infection prevention and control practices are inadequate knowledge and incorrect attitude among healthcare workers.^9,10^ Various studies worldwide have identified significant gaps in the AMR component in pre-service and in-service curricula for health professionals.^9,11,12,13^ A survey conducted between 2018 and 2019 using self-administered questionnaires found that only 36.6% of final-year medical and pharmacy students from three universities in Uganda, Kenya and Tanzania were adequately trained about AMR and antibiotic use in clinical scenarios.^14^ As a strategy for strengthening human resource capacity for AMR containment, the WHO Global Action Plan on AMR recommends integrating AMR into professional education, training and certification of the One Health workforce.^15^ To guide this process, WHO developed a competency framework for health workers’ education and training on AMR.^16^ A recent analysis of Uganda’s school educational curricula revealed a significant deficiency in basic AMR principles and health security across all education levels. Students under 16 years receive less than 40% of the necessary content. There is a complete absence of content on rational antibiotic use, health-seeking behaviours and One Health, highlighting the need to incorporate additional AMR content and undertake policy reviews to address these gaps.^17^

There is an urgent need to enhance awareness, education and training to improve the knowledge and attitude of healthcare professionals on AMR. This aligns with the first strategic objective of the WHO’s Global Action Plan and Uganda’s National Action Plan for AMR.^15,18^ Efforts must extend beyond addressing the molecular and biomedical aspects of AMR to also address its broader societal implications.^19^ This can be achieved at both the pre-service and in-service levels. Enhancing pre-service training for health professional students is a cost-effective and sustainable approach to improving health worker knowledge, skills and attitudes toward AMR and antibiotic use.^19,20^ Additionally, interventions targeting school children have been shown to effectively improve their knowledge and understanding of AMR and influence their parents, families and communities towards appropriate antimicrobial use.^21^

In addition to providing students with comprehensive knowledge and understanding, there are opportunities to engage them as active participants in practical actions both within and outside the school environment. These action-oriented initiatives can reinforce what students have learned through classroom settings and motivate them by offering opportunities to apply their knowledge in real-world contexts. This hands-on approach helps students to develop essential skills and attitudes crucial for AMR containment, which are often weak components of traditional education, particularly in low- and middle-income countries.^21^

The US Agency for International Development (USAID) Medicines, Technologies and Pharmaceutical Services programme (henceforth called ‘the programme’)^22^ has been supporting Uganda’s efforts to combat AMR, engaging with diverse groups of stakeholders, including the national multisectoral AMR governance bodies, regulatory authorities, facilities’ managers and care providers, the private sector, universities and health professional students. Students have not been fully leveraged in AMR containment, despite their position as future healthcare practitioners, implementors and decision-makers for sustaining ongoing efforts including the USAID’s local capacity strengthening and localisation agenda.^23,24^ This article describes a multi-pronged process to collaborate with various Ugandan entities in building a team of future health professionals dedicated to leading a sustainable fight against AMR.

## The multi-pronged process

Students’ engagement aimed at involving students in practical actions to enhance their knowledge about AMR, emphasising its burden and their role in containment initiatives. This was achieved through an organised, multi-pronged, multi-step approach described further in the text.

**FIGURE 1:**
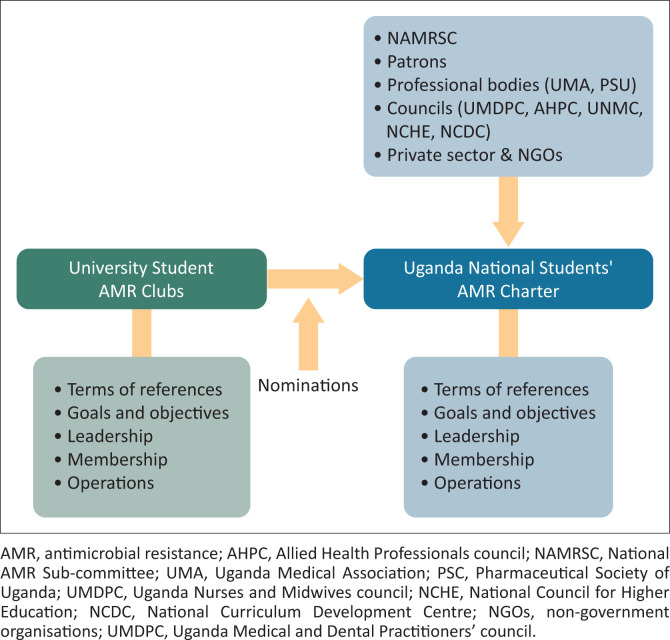
The placement of the student’s antimicrobial resistance Charter in the national structure.

The efforts started with a collaboration between the Pharmaceutical Society of Uganda, the programme and the Uganda National AMR Sub-committee (NAMRSC), which is the national-level multisectoral governance body overseeing the operationalisation of the country’s National Action Plan for AMR.^18^ Each of the three entities – one being a professional association, one serving as a donor funded implementing partner and one being the national coordinating body on AMR – brought their complementary strengths to the initiative as partners aiming to improve awareness, knowledge and training on AMR among health professional students. The Pharmaceutical Society of Uganda has an Educational Committee and an AMR Committee both with the mandate to educate the population about AMR. They were already implementing activities for increasing AMR awareness among pharmacy students. Therefore, the collaboration leveraged the Pharmaceutical Society of Uganda’s expertise to include students from other health professions. This jump-starting collaboration led to a series of subsequent actions and stakeholder engagements, culminating in the formation of a National Students’ AMR Charter (Figure 1). In Uganda, there are currently 18 universities licensed to train future healthcare professionals for both human and animal health. Of these, we supported seven but with a vision to roll-out to all universities through the national structure. The universities were purposively selected for three reasons: (1) they are the most populous ones in terms of the students enrolled; hence, we anticipated a bigger impact given the potential for reaching out to a large number of the students, (2) they are distributed in different regions of the country, thus creating a potential for them to coordinate with other universities in their respective regions and (3) they had students at all years of study, unlike some other universities that were in their early years of establishment and thus had students only within the first few years of study.

### Conducting symposia on antimicrobial resistance for university students

The Pharmaceutical Society of Uganda, NAMRSC, and the programme collaborated with universities to organise seven 1-day symposia on AMR in each of the seven supported universities. The overarching purpose of the symposia was to generate interest in AMR among students as the initial step in setting up university-based student interest groups that would coordinate AMR activities. The symposia targeted students in the human, environmental and animal health-training faculties and departments, including medicine, pharmacy, pharmaceutical sciences, nursing, medical laboratory sciences, veterinary medicine and environmental health sciences. Authorisations for the symposia were sought for and obtained from the faculty heads. Students were informed about the upcoming symposia, and they voluntarily attended the symposia. The symposia were held on university premises, with key speakers from the faculties, departments, the programme and the Pharmaceutical Society of Uganda; all of the speakers at least had a postgraduate degree and experience working in the field of AMR research, academia or programme implementation. The topics of discussion included an overview of antimicrobials, burden of AMR, causes and mechanisms of AMR, drivers of AMR, the One Health concept, strategies for AMR containment, best practices, infection prevention and control and the role of students in AMR containment. There were opportunities for students during the symposia to share stories about what they had witnessed and heard about AMR, including challenges, which were extensively addressed by the key speakers. During these discussions, the students also provided significant feedback on the organisation of the symposia and the next steps. While giving their feedback, many participants highlighted the impact of the symposia (Table 1). An average of about 12 million Ugandan shillings ($3300.00) was invested in each symposium with a total attendance of 1000 students in all the seven university symposia.

**TABLE 1:** Select quotes from different participants attending the antimicrobial resistance symposia.

Quotes	Participant
‘Yes, together we can defeat AMR. We can revert this impending trouble back to normality. It begins with us all.’ (Medical student, Busitema University, Mbale)	Medical student
‘Medical practitioners need to understand that they can’t break away from the ecosystem, we must collaborate. A holistic approach is vital here.’ (Environmental Health Sciences student, Makerere University, Kampala)	Environmental Health student
‘Allow me to express my profound gratitude to the organisers, presenters and everyone that attended this informative symposium. It looks like the AMR problem is bigger than I thought, and we all need urgent response to it.’ (Nursing student, Busitema University, Mbale)	Nursing student
‘Thanks for enriching us with knowledge on AMR. I’m left not the same as I was yesterday; looking forward to learning more.’ (Veterinary Medicine student, Makerere University, Kampala)	Veterinary Medicine student
‘This is a very important symposium for us all; today we are bringing together our foot soldiers as far as AMR stewardship is concerned … it is important that they know the global challenges before they graduate and the challenges in Uganda as key stakeholders. As a country, we are realising the importance of One Health concept.’	Dean from a university

### Establishing university students’ antimicrobial resistance interest groups

Riding on the momentum and interest generated from the AMR symposia, the NAMRSC, the Pharmaceutical Society of Uganda and the programme facilitated establishment of AMR interest groups with the goal of sustaining conversations resulting in specific planned actions that the students can own. To form these groups, the faculty members reached out to the students after the symposia and invited those that had interest in further engaging in AMR containment activities to get in touch with them. Among the students that reached out to faculty members, tentative students’ leaders were selected by the faculty members to lead the formation of the groups. The programme supported a series of workshops that brought together the students that had demonstrated interest in AMR and were designated as ‘AMR champions.’ The students voluntarily opted and were supported to establish university AMR interest groups (or ‘clubs’) under the guidance of a faculty-based AMR expert designated as a patron. During these workshops, each costing about 4 million Ugandan shillings ($1100.00), the state of student AMR activities was analysed, terms of reference for the AMR clubs were drawn up and adopted and an interim leadership committee was formed to coordinate AMR club activities. The terms of reference established the leadership committee composition and detailed their roles, centred around raising interest in the fight against AMR and increasing awareness among fellow students and their neighbouring communities. The students then elected leaders for their respective university groups. The students’ leadership was supported through the patron to register club members, undertake selected activities and share reports on the status of the clubs.

The clinical and academic mentors (patrons) for each club were identified and were responsible for providing technical and operational guidance to the AMR clubs, receiving feedback from the students and linking the students’ clubs with the university administration. In addition, the AMR clubs nominated two members of the leadership committee to the National AMR Students’ Charter. Subsequently, the AMR clubs developed work plans with activities focusing on increasing AMR awareness among students, faculty members, patients and members of the surrounding communities. Together with the patrons, the clubs were launched and incorporated into existing university structures. The university group leaders working with other AMR champions continued to rally other students to join them in several activities, and from these activities, more students were registered to join the club, which saw the clubs grow.

The programme provided financial and logistical support to the student interest groups to organise activities like regular grand rounds, journal clubs and community outreaches and created discussion forums to share knowledge and discuss key issues on AMR, ranging from case reports, case series, diagnostic laboratory findings and journal articles. On various occasions, technical personnel from the programme were key speakers and facilitators on the events organised by the students’ groups.

### Uganda National Antimicrobial Resistance Students’ Charter

Following the successful formation and solidification of individual university students’ AMR clubs, the key players involved in the initiative embarked on consolidating, coordinating and institutionalising the efforts to form the National AMR Students’ Charter, which would coordinate nationwide efforts and bring other institutions on board. To foster this, the university AMR clubs’ chairpersons and patrons, NAMRSC, and Department of Clinical Services at the Ministry of Health, and other stakeholders held joint consultative meetings. The meetings aimed at sharing the progress made on pre-service One Health professional student engagements, projecting the future scope and impacts of the institutional AMR clubs and discussing the need to have a formal coordination mechanism for the AMR clubs at the national level by establishing the National Students’ AMR Charter.

**FIGURE 2:**
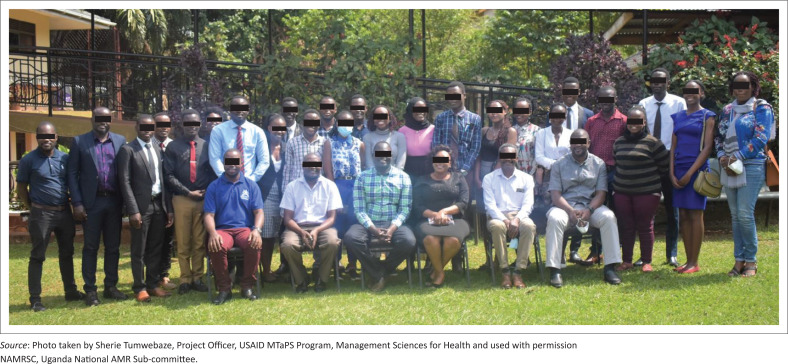
The leadership of the students’ antimicrobial resistance charter together with their patrons, the chairperson of the NAMRSC and the commissioner for clinical services at the Uganda Ministry of Health.

**FIGURE 3:**
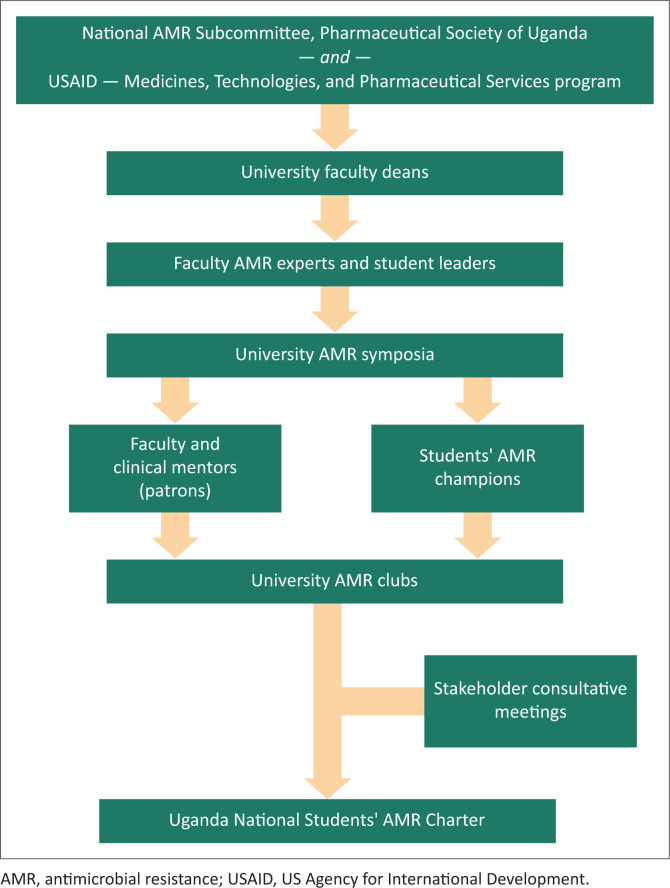
The steps taken to establish the Uganda National Students’ antimicrobial resistance Charter.

Terms of reference were drafted for the charter and shared among the different stakeholders and interest groups for reviews, discussions and inputs. The NAMRSC, Department of Clinical Services at the Ministry of Health, and the students’ interest groups represented by their team leads and patrons then convened a meeting, which agreed on some key structural and functional arrangements for the charter. Those included (1) the placement of the students’ charter in the national structure (Figure 1); (2) the name of the charter (Uganda National Students’ AMR Charter); (3) the patron to the charter who would be the chair of NAMRSC; (4) the terms of reference and (5) selection of student leaders for the charter (Figure 2). The National Charter was then launched by the chairperson of NAMRSC, who was mandated to coordinate AMR-related activities in all tertiary institutions. An advisory board composed of the club patrons and representatives from professional bodies was also established to act as the link between the Uganda National Students’ AMR Charter and NAMRSC. The advisory board also receives feedback from the students’ charter and provides guidance. The National Charter was structured to involve other universities, in addition to the original seven universities, and is expected to be continuously supported by NAMRSC to execute its mandate to contain AMR by creating awareness among the future health professionals. For this launch, a total of about 30 million Ugandan shillings ($8300.00) was invested to bring together university club leaders and other stakeholders. This process is summarised in Figure 3.

## Quality assurance measures

To ensure that the students received the most relevant content delivered to them during the symposia, a comprehensive review of the curricula content was conducted with a variety of stakeholders including academicians, members of professional bodies and technical members of the programme. The review identified essential but missing content and incorporated the latest advances in AMR containment to develop the symposium materials. Additionally, the terms of reference for both the university groups and the National Charter were reviewed by various stakeholders to ensure they effectively guide students and enable the groups and the charter to contribute meaningfully to AMR containment efforts. Lastly, programme technical personnel and group patrons provided ongoing guidance to ensure the quality and impact of the students’ activities in combating AMR.

## Progress on advancing student-led antimicrobial resistance efforts

**FIGURE 4:**
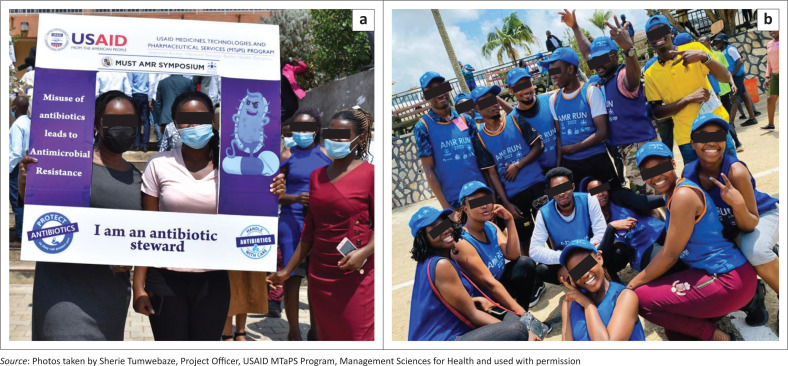
(a and b) Students from the Faculty of Medicine of Mbarara University of Science & Technology.

Today, Uganda holds a forum that brings together students interested in fighting AMR. The Uganda National Students’ AMR Charter has a structure that interlinks with NAMRSC, the professional bodies and universities. This approach has helped formalise and mainstream the activities spearheaded by pre-service students, recognising these stakeholders as important allies in AMR containment efforts. The interest in the fight against AMR has grown over time, evidenced by subsequent initiatives the students have undertaken since institutionalisation of both the interest groups and the National Charter. These activities include patient education on hospital wards, grand rounds on AMR, publications on AMR,^25,26,27^ applying for competitive AMR-related research grants, short online videos,^28^ physical and virtual webinars, radio talk shows, social media campaigns,^29^ community walks (and carrying placards with information on AMR) and runs for AMR (Figure 4), contributing articles to the biannual Uganda One Health antimicrobial stewardship newsletter,^30^ among others.

The increased visibility of students involved in the AMR clubs has brought them opportunities for mentorship as members of faculty research groups or as interns on big research projects on AMR. In clubs where this occurred, a ripple effect was created in the student clubs as the mentored AMR champions then transferred their knowledge and skills to their colleagues, inspiring them to apply for small grants of their own. Eight out of 12 applications that were submitted by the mentored students were successful. Five students received prizes while three student teams received grants estimated at $173000.00 from both local and international funders (Table 2). The symposia brought together 1000 students, of which 63.3% were male and 36.7% were female (Table 3), from different professions and universities distributed all over the country, learning about AMR, addressing major knowledge gaps. Within a period of one and a half years from the inception, the charter has supported six additional universities to form their own interest groups, and the total number of registered students has grown to 2350. With this, the programme working with the Pharmaceutical Society of Uganda, NAMRSC, and other key stakeholders, has significantly contributed to addressing both the global and national action plans’ strategic objective 1 (awareness and understanding of AMR).

**TABLE 2:** Grants received by students’ teams.

Awarding organisation	Grant title	Amount (USD)	Outcome
ReACT Africa	Knowledge, attitude and practice towards AMR among medical interns in Uganda	1000.00	Completed and published†
Pfizer	Strengthening nurses’ capacity for antimicrobial prescription in lower health facilities in Uganda Study	73000.00	Ongoing capacity building for nurses
Pfizer	AMS and IPC in the Intensive Care Unit at Mbarara Regional Referral Hospital	99000.00	Ongoing research

AMR, antimicrobial resistance; USD, united states dollar; IPC, infection prevention and control.

† , Nabidda S, Ssennyonjo R, Atwaru J, et al. Antimicrobial resistance and rational prescription practices: Knowledge, perceptions and confidence of health profession interns in Uganda. JAC-Antimicrob Resist. 2023; 5(5):dlad105. https://doi.org/10.1093/jacamr/dlad105.

**TABLE 3:** Students that attended the university symposia.

University	Number of students (*n*)		
	Female	Male	Total
Makerere University	138	302	440
Mbarara University of Science and Technology	44	106	150
Busitema University	79	68	147
Kampala International University	72	109	181
Islamic University in Uganda	18	29	47
Kabale University	12	23	35

**Total**	**363**	**637**	**1000**

Subsequently, a joint review of the undergraduate curriculum competencies on AMR by the respective universities and professional bodies, with support from the Uganda National Council for Higher Education, was undertaken. This was intended to assess whether the specified content, learning outcomes and method of delivery address the competence needs of the graduates to tackle the challenges faced with the growing AMR burden as outlined in the WHO competency framework for health workers’ education and training on AMR.^16^

To conduct the review, the Uganda National Council for Higher Education collaborated with various stakeholders, including professional bodies, the Ministry of Education, learning institutions and the National Curriculum Development Centre, during a 5-day residential workshop. Portable Document Format (PDF) copies of curricula from different universities for all medical courses were obtained and systematically searched for content related to AMR. Following this review, several gaps were identified in all the curricula and new AMR training content was developed in consultation with the schools, professional councils and other stakeholders, including the National Curriculum Development Centre to address these challenges. Moving forward, the National Council for Higher Education will assess if the medical schools’ curricula contain the new content on AMR during the next round of submission for curriculum review by the learning institutions. There is now a clear channel of pre-service health professional students that the government and other groups can collaborate with to champion activities against AMR.

## Challenges and lessons learned

Our work faced significant challenges throughout its execution, primarily stemming from government-imposed restrictions on mobility and in-person interactions because of the escalating COVID-19 infections within the population.^31^ Consequently, we resorted to virtual means to continue engaging the AMR champions and their patrons and provided strategic direction in organising the activities. Further complicating the situation, poor internet connectivity among university students hindered their capacity to engage fully and effectively in virtual group activities. The programme supported the designated student AMR champions with mobile data bundles for meetings to facilitate continuity of the activities. We emphasised that the AMR champions who were successful in attending the virtual engagements should cascade the information to the rest of the members of the AMR clubs. Additionally, the involvement of multiple stakeholders during the planning and implementation state proved to be more time-consuming than expected, as some of them could not respond promptly because of high workload and competing priorities. Subsequently, we adopted a significantly more proactive approach, initiating much earlier planning and persistent stakeholder engagement to ensure that we could make progress in a timely and efficient manner. Lastly, given inequities in funding and limitations in existing infrastructure, Uganda and other countries must prioritise such activities accordingly, allocate sufficient budget and leverage other resources for successful implementation.^32^

Some of the lessons learned through our work include realising that there exists a lot of potential in students to conduct a range of activities that would help accelerate the impact of interventions if students are engaged in such activities. The students in different university groups conducted ward-based patient education and community engagement activities reaching a wider range of communities that the programme would not reach in such a short time. This also means that engagement of students to cascade practices would increase the multiplier effects by enabling the proposed intervention to reach a wider number of beneficiaries in a short period of time.

Furthermore, our work showed that pre-service health professional students are interested, available and can be effectively engaged to support health promotional activities. This provides an opportunity to give birth to a new community of practice to support AMR containment and other health interventions.

## Future directions and recommendations

The article presents the establishment of institutionalised and sustainable structures and systems in Uganda that champions the role of students in the fight against AMR. This systematic approach can serve as a solid model to further engage and enhance the roles of current and future health professional students across the human, animal and environmental sectors, contributing to the spirit of One Health. Health professional students and other groups, such as school children, journalists, civil society organisations and consumer groups, should be increasingly mobilised to join hands with the traditional mainstream stakeholders to fight the multi-faceted problem of AMR, thus widening the stakeholder base and contributing to the whole-of-society engagement. Students can be supported to undertake activities out of their routine academic syllabus, like panel discussions and AMR campaigns.

Championing the already available stakeholder group of students by raising their interest and engagement in practical actions can be an additional effective, affordable, easily replicable and sustainable approach in addressing AMR. Moreover, such action-oriented engagements can create a practical frame of reference in the minds of pre-service students for future continuity in practice after completing their studies and joining the workforce. This approach has been suggested as key in the fight against AMR as early engagement can provide a strong basis of AMR knowledge that students can benchmark on as they progress through their careers as prescribers and patient educators.^20^ The knowledge and skills gained during this learning period provide students with the necessary competence and confidence for practice that could preserve the efficacy of antimicrobials.^19^ Students have also been easy to get interested with the fight against AMR compared to already in-service health professionals, and thus provide a new forum for AMR awareness. Students are future health professionals in training and thus if this changes their attitudes and improves their knowledge, it can impart positively on the fight against AMR.

Because AMR is recognised as a global health security threat, this approach can be replicated in other countries and be applied to the wider global health security human resources strengthening efforts by governments and implementing partners supporting national health security programmes.

We recommend that governments in low- and middle-income countries and implementing partners undertake comprehensive reviews of existing in-country curricula to ensure they align with the required competencies for AMR containment and other global health security concerns. This should be followed by developing and/or updating curriculum competencies at all educational levels. These competencies should extend beyond classroom instruction to include extracurricular activities as well.

## Conclusion

This article documents a process that was used to raise pre-service students’ interest in the fight against AMR, leading to the formation of a national team of student AMR champions, and how this process can be embedded into the national system, systematically contributing to the strategic objective 1 outlined in the WHO Global Action Plan on AMR. This Ugandan experience showed that the national multisectoral governance bodies on AMR, professional associations and university faculty members are willing to embrace the value of and collaborate with health professional students in widening approaches to operationalising the national action plan on AMR. This experience of engaging and collaborating with existing students can potentially be replicated as a simple, institutionalised, sustainable and affordable approach in other low- and middle-income countries for a sustainable fight against AMR. The approach also serves as an avenue to break the traditional silos of different disciplines and contributes to One Health by bringing human, animal and environmental sector students together to carry out joint and collaborative actions against AMR.
